# A knee monitoring device and the preferences of patients living with osteoarthritis: a qualitative study

**DOI:** 10.1136/bmjopen-2015-007980

**Published:** 2015-09-07

**Authors:** Enrica Papi, Athina Belsi, Alison H McGregor

**Affiliations:** 1 Department of Surgery and Cancer, Imperial College London, Charing Cross Hospital, London, UK; 2 Department of Surgery and Cancer, Imperial College London, St Mary's Campus, London, UK

**Keywords:** Focus group, Rehabilitation, Osteoarthirits, Functional monitoring, wearable system

## Abstract

**Objectives:**

To identify perspective of patients with osteoarthritis, in particular design requirements and mode of use, of wearable technology to support the rehabilitation pathway. This study is part of a user-centred design approach adopted to develop a rehabilitation tool for patients with osteoarthritis.

**Design:**

Qualitative study using a focus group approach; data management via a thematic analysis of patients’ responses.

**Participants:**

21 patients with osteoarthritis (age range 45–65 years) participated in 1 of the 4 focus groups. Recruitment continued until data saturation.

**Setting:**

The study was conducted in a university setting.

**Results:**

Main determinants of user acceptance of a wearable technology were appearance and comfort during use. Patients were supportive of the use of wearable technologies during rehabilitation and could recognise their benefit as monitors for their progress, incentives to adhere to exercise, and tools for more informed interaction with clinicians.

**Conclusions:**

This paper should encourage adoption and development of wearable technology to support rehabilitation of patients with osteoarthritis. It is pivotal that technological development takes into account patients’ views in that it should be small, light, discrete, not ‘appear medical’ or challenge the identity of the user. Derived data should be available to patients and clinicians. Furthermore, wearable technologies should be developed to operate in two modes: for exercise guidance and assessment only, and for unobtrusive everyday monitoring. The information obtained from this study should guide the design of new technologies and support their use in clinical practice.

**Strengths and limitations of this study**

- Focus groups permitted us to have an in-depth insight into patients’ views on the use of wearable technology.
- Patients with osteoarthritis from diverse socioeconomic background participated in the study.
- Participants were not able to test the technology.
- This paper presents patients’ views of wearable technology which to date have largely been ignored in technology development and therefore, accounts for the low uptake of these technologies.

## Introduction

Wearable technologies, defined as portable devices that can be embedded in the user’s outfit as part of the clothing or an accessory, enable data gathering—mostly related to health and fitness—over extended periods of time, unobtrusively.^1^ ^2^ Recent advancements in miniaturised electronics, in parallel with the growing numbers of the technologically adept among our population, have fostered an increased interest in wearable technology and their use for clinical purposes. This is further supported by the recognised benefits derived from long-term monitoring of patients in real-life environments, and the predicted reduction in healthcare costs following adoption of such technologies.^1^ ^2^

Rehabilitation based on exercise therapy is recommended for people living with osteoarthritis (OA).^3^ Exercising conveys benefits for patients, including reduced pain, enhanced joint function and quality of life.^4^ Patients with OA however are reluctant to adhere to prescribed rehabilitation programme over long time periods, thus compromising and limiting the benefits of this intervention.^5^ Adherence increases during supervised exercise sessions, but delivery of these is economically resourceful.^6^ Among the reasons leading to poor adherence, the majority are related to organisational issues such as time and locations concerns, and conflicts with everyday commitments.^6^ Psychosocial issues, poor motivation and lack of understanding of the rehabilitation content and perceived benefits further affect adherence.^6^ ^7^

Wearable technology gathering information relating to patients’ functions could potentially be used to provide feedback on accomplished goals in rehabilitation and inform treatment to maximise the benefit of care according to each individual's specific need. Moreover, being portable, these will allow patient monitoring and guidance during exercise in their chosen environment overcoming organisational barriers to adherence. Despite the numerous wearable systems introduced in research scenarios, clinical adoption remains poor.^8^ Most of the studies conducted to date have focused on the validation and use of wearable technology in the laboratory environment.^8–10^ Moreover, the systems were confined to analysis and comparison of movement patterns between healthy and pathological populations with only few using the acquired data for feedback to patients and application in clinical practice.^8^ When wearable technology was used to measure complex descriptors of human movement, such as joint kinematics variables, usually acquired with laboratory-based equipment,^11–13^ the systems used were cumbersome and difficult to operate by non-experts. Researchers have focused mainly on the engineering aspects of the technology with users’ preferences receiving little attention.^14^ This in part explains the mismatch between the number of available technologies and their clinical adoption. There are questions that remain unanswered. For example: (1) Do the measures collected and analysed within the research practice go beyond their mathematical correctness of easy interpretation for clinical use? (2) How can these measures be employed by patients or clinicians in the management of disabilities? (3) How can additional information change patients’ attitude towards rehabilitation regimes? (4) How would they like the extra information to be utilised? (5) Which form of feedback is preferred? (6) How long would patients wear such a device? (7) What should it look like?

The few studies that have explored patients requirements have identified that patients want systems to be small, with minimal interference with their everyday tasks, and be easy to use.^14–16^ Few if any of the technologies developed to date reflect these requirements. Patients’ and health professionals’ preferences should be an integral part in the design process of the technology for it to progress into clinical practice and ultimately lead to patient benefit. The questions reported above should be rigorously explored at an early stage of the design process.

We, therefore, have adopted a user-centred approach while developing a wearable technology to monitor knee functional status in patients affected by OA. The technology, in its prototype form,^17^ is characterised by a small flexible polymeric conductive strip embedded into a pair of leggings. Small wearable electronics are connected with the flexible sensor to allow wireless data acquisition. To foresee clinical translation of our technology and finalise its design, we discussed with patients their views, preferences and expected use of the technology. This paper articulates the requirements for the design process. Although, this paper focuses on a particular technology, our custom built wearable system, it also allows generalisation of the findings to be applied to the design of wearable technology for rehabilitation purposes, particularly in relation to the patients’ intended use.

## Methods

This was a qualitative study using focus groups to investigate patients’ perspectives of wearable technology. All participants provided written informed consent prior to taking part in the study. Patients were recruited from the Imperial College NHS Trust physiotherapy departments and local communities via poster advertisements. Twenty-one adults (19 females, 2 males, age range 45–65 years) suffering from OA volunteered to take part in this study. Participants were sampled based on being diagnosed with OA through clinical assessment or imaging, undergoing rehabilitation, and having a good understanding of written and spoken English. They were excluded from the study if they presented with neurological conditions that may have influenced their cognitive function.

Each patient participated in one of four focus groups, which took place in a quiet room of the Imperial College London, Charing Cross Campus. The time duration for each focus group was between 45 and 60 min. Two moderators (AB and EP) facilitated the discussion by following a semistructured topic guide. Box 1 shows the discussion flow stream with some associated questions. Each focus group began with an introduction clarifying the format of the discussion and assuring the confidentiality of the information exchanged. The aims of the study were thoroughly described; the definition of wearable technology and description of the prototype developed was provided. The prototype of the flexible sensor unit and electronics components were shown to the group. The debate could then be articulated following topics in box 1. Each focus group was audiorecorded and verbatim transcribed to allow subsequent analysis.

Box 1 **Focus group discussion semistructured topic guide**

1. *Introduction*
  - Moderators and participants introduce themselves
  - Clarification on the format of the focus group and aim
  - Assurance of confidentiality
2. *Wearable technology*
  - Definition of wearable technology
  - Ask if they know of any wearable devices and demonstration of prototype developed
  - Ask if they like this kind of technology and if so why:
    Would you use it?
    How often will you be willing to wear it? Daily?
  - Ask what they do not like about this kind of technology and if so, why:
    What would put you off in using such technologies?
3. *Feelings about wearable medical technology*
  - How are you doing in general in dealing with your disease?
  - Do you think wearable technology would help your current situation? If so, how?
  - How do you view this technology in comparison to conventional forms of treatment? And why?
  - Do you see yourself using this kind of technology? If so, how?
  - Would you use this technology to monitor your rehabilitation practice in your home rather than going to a clinical practice to attend rehabilitation classes?
4. *Impact on relationships*
  - If you did decide to use this technology, how do you think it would impact on your daily interactions with others?
  - Do you think it would change how you interact with medical professionals?
  - Do you think it would affect your home life/working environment (if applicable)?
  - What are your views on data privacy?
5. *Closing*
  - Is there anything else you would like to say about what we have discussed?
  - Thank everyone for their time and useful participation

A thematic analysis was conducted on each focus group at respondent level using Framework Methodology.^18^ Data analysis was conducted separately by the two moderators for cross-validation of the outputs from each focus group before grouping the results. Key themes were identified from which concepts could be developed. These were used for comparison among focus groups, and for data mapping and interpretation. Data saturation was reached while analysing the fourth focus group; hence, recruitment was ceased. Classification of patients’ responses in the different themes and concepts identified was done in Microsoft Excel spreadsheets.

## Results

Only a few patients (9/21) were aware of what wearable technology was and could provide valid examples of such systems. Providing a comprehensive explanation about wearable technology and showing them the prototype we developed allowed us to proceed in the discussion on their views and preferences regarding this technology.

**Figure 1:**
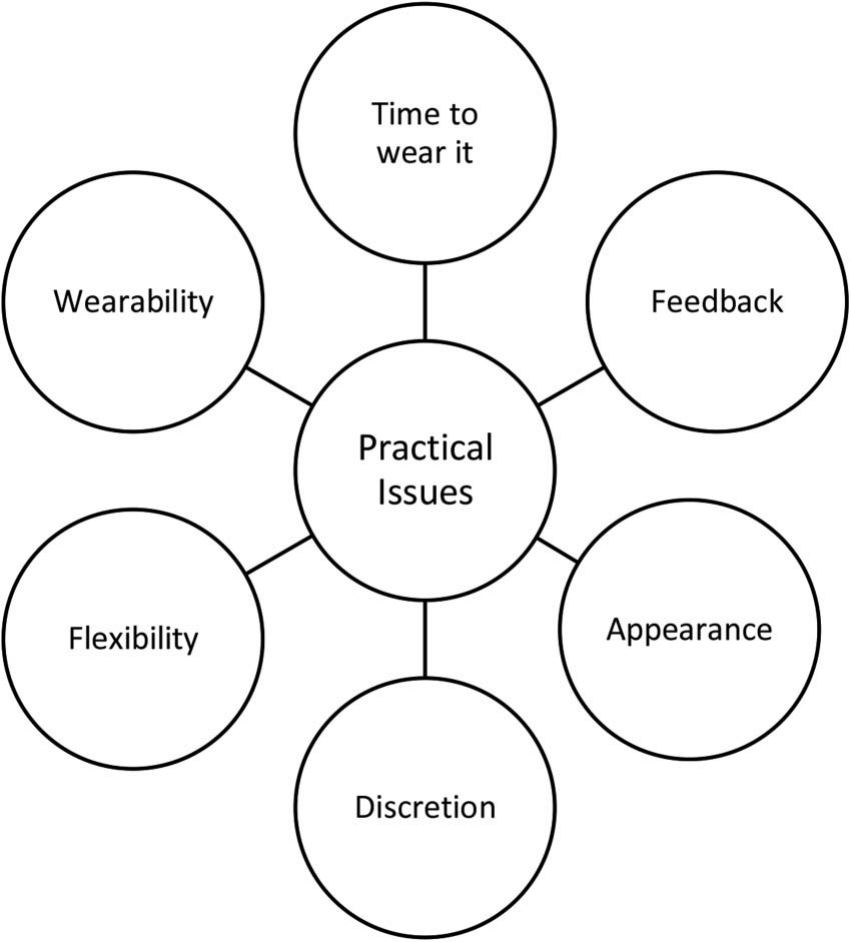
Patients’ views on practical issues.

**Figure 2:**
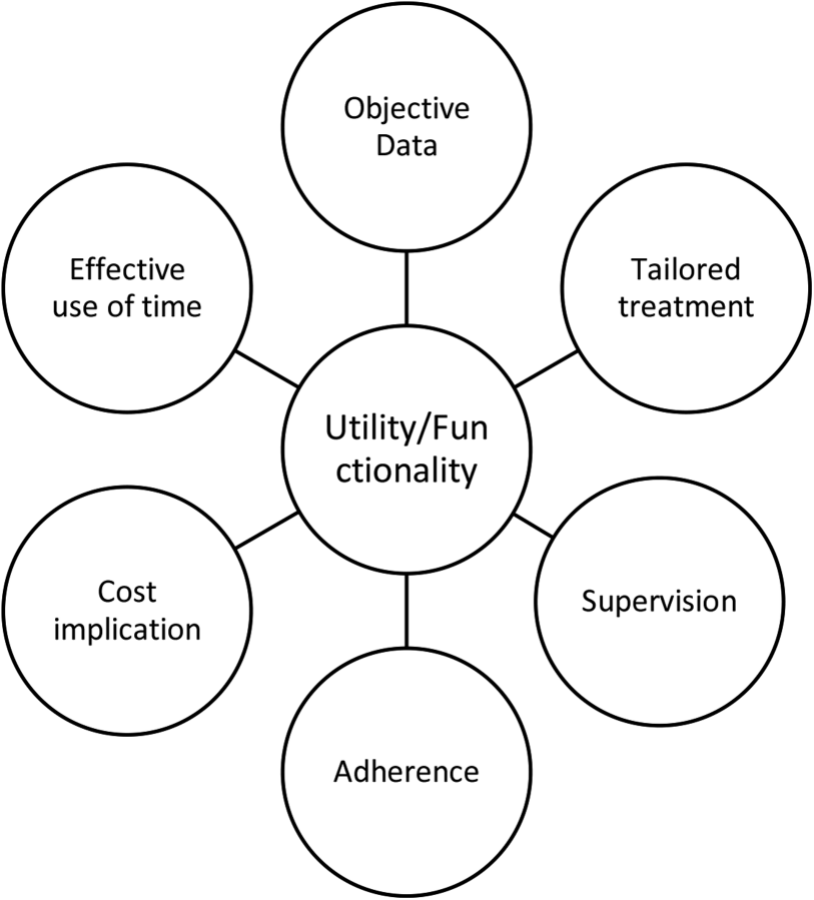
Patients’ views on utility/functionality.

The focus groups revealed recurrent concepts as expressed in the participants’ views. The findings suggested five overarching themes patients associated with wearable technology, which are linked and intertwined: *practical issues, utility/functionality, patient–doctor communication, social impact and empowerment*. Given the aim of this paper, which looks into design requirements for our wearable system, only the first two themes will be discussed in detail. *Practical issues* and *utility/functionality,* along with their associated concepts, are presented in figures 1 and 2.

Where quotes are reported the acronym FG followed by number 1–4 is used to indicate the focus groups, and F or M indicates gender, female or male, respectively, of the participants at the focus group they attended.

### Practical issues

Patients associated wearable technology with practical issues and the impact of these on their life. The views expressed encompassed issues regarding sensor wearability, appearance, *when to* and *how long to* wear the device, comfort and design, as well as discreetness and privacy.

All patients expressed a positive attitude towards wearing our wearable technology, although different views emerged on how long they would be willing to wear it. For instance, some participants were willing to wear it only for few hours per day, particularly when exercising:

I could envisage wearing it for a few hours a day, like when I was specifically doing exercises or something that was giving a lot of feedback either to me or to the clinician, but I'm not sure that I would be happy wearing it all day everyday (FG2, F1)

However, some would also wear it over prolonged periods, as they acknowledged the advantage of a real situation monitoring:

It would be good information for the clinical team to get that[data collection], even when we're just walking down the street or whatever (FG3, M1)

It's when you're actually out, trying to get on the bus or trying to get off the bus, that you really find that your weakest bits are going and so that would be able to identify some of those. So yes indeed, I would, is the answer to that (FG3, F1)

While wearing preferences during the day varied, all patients agreed that they would not wear the device at night and when relaxing.

Patients also discussed their preferences in the way wearable technology could be embedded in the clothing. An issue patients noted with the current prototype was related to the integration of the sensor onto a pair of leggings and the unlikelihood of wearing leggings throughout a day. Rather, they stated the preference of having the sensor integrated into a band to be positioned around the knee:

I would prefer a band, something that's simple, just put it on quickly, take it off quickly, and get on with it (FG4, M1)

Different design options which would accommodate and fit better with their lifestyle were also discussed:

Do you think a distinction should be made between something you wear for exercise as opposed to something you'd wear all day because you're going on a walk? I guess people would be willing to put certain things on as part of exercising as opposed to day to day, all day (FG1, F4)

Other patient preferences, however, had to do with skin colour and having the option of wearable technology to match it, so that it would be less visible and more discreet:

Personally I don't see it as a feature so I would want mine to be a nude colour. I'm not wearing it because I want it to be seen. I'm wearing it because I want to know how my knee's doing (FG4, F2)

The older participants were not concerned on the appearance of the technology, a finding which suggests that age played a role in their views:

I don't think in our ages orange fluorescent or spotted or striped will make a difference we don't need a fashion item (FG1, F4)

When discussing day-by-day use of the technology, among the factors that would discourage patients to wear it would be if the system is ‘*uncomfortable*’, ‘*itchy*’, ‘*hot*’ and if it ‘*buckles up*’, ‘*bulges out*’ and ‘*moves around*’, suggesting that a small size and weight would also facilitate acceptance:

I think weight would be a major factor as to whether, how long you could cope with it (FG2, F3)

If it's something small you're much more likely to wear it all day (FG4, F2)

Flexibility in the choice of clothing was another important issue highlighted in patients’ views, as they would prefer to have a choice in what they wear, rather than having to put on a specific clothing item just because the sensor would be attached to it. For this reason, having the sensor embedded in leggings was disliked:

I wouldn't want it in a pair of leggings because I wouldn't want you determining what I wore. I would want it as a band and then I'd wear what I want. It gives you flexibility as to what you wear it (FG4, F2)

It was raised that specific clothing could be identified as ‘patient clothing’, thus bringing in concerns of being ‘labelled as patients’. Identity intrusion seemed to be an important demotivator:

I think if it's in a pair of leggings automatically you have become a patient. Do you know what I mean? You've become medicalised, whereas if you're just wearing a band then you're you with a band. It's a different thing and you keep your identity with the band (FG4, F3)

Both discreteness and maintaining one's privacy were, therefore, a major concern in the decision to wear a technology or not:

If you have something that draws everybody's attention to it you're going to be questioned about it and, to be honest with you, I don't really want to go through my medical history with the world (FG4, F1)

Particularly, the appearance could bias their appreciation of the system:

If it looked too medical I would be less happy wearing it (FG2, F1)

Patients, hence, suggested paying attention on the design to avoid the stereotype of a medical item but without looking ‘*too out of the ordinary*’:

If it looked quite nice, it is a piece of clothing, I'd be much happier wearing it than if it looked like a kind of medical thing (FG2, F1)

There were also suggestions that the sensor should provide haptic feedback in the form of vibration or little impulse “as a little reminder to telling you you're not walking in the correct way, come on, get it right, you'll find it easier in the long run” (FG2, F3). However, in doing so, it should remain “silent” so as to not “draw attention to yourself in anyway” and hence, be discreet.

### Utility/functionality

The majority of participants recognised the benefits of being monitored to obtain objective data on their joint functional status and it was acknowledged that this would be useful for themselves as well as for their clinicians. Their responses connected the use of wearable technology with issues such as the advantages of constant and objective monitoring, the impact that using this data would have on adherence and compliance, as well as managing their condition and reducing the relevant costs. These issues are developed below.

In terms of themselves using the data gathered with the use of wearable technology, patients were positively keen in obtaining more information on their condition so that they could observe their progress, monitor their status and guide their actions:

It would be very nice to have something which could actually inform you as to what you might be doing, how you might be moving incorrectly and how to correct that problem to really stop it before it becomes an issue (FG4, F3)

Interestingly, it was perceived that having clearer information about their health would motivate them to comply in a consistent way to treatment and improve their condition:

The thing is if we know we're doing something right, we're going to progress so much better, aren't we? (FG2, F3)

The participants also talked about adherence, as there were some who more directly saw the device as a way to adhere more with the exercise regimes once the supervised sessions ceased, which was also facilitated by the ability to perform exercise at home:

I do them[the exercise], only because over the years I have now learnt that if I don't it gets very much worse. If I'd had something like that to prompt me ten years ago when I didn't do my exercises, I probably would have done them more and it would have been better. I may not have, but I have now become quite diligent, so that's OK, but I think it might have helped to have had something like that when I was less diligent (FG2, F1)

So we can do it at home. We can do it by ourselves really (FG3, F2)

The technology was seen as an alternative way to provide supervision when away from a clinical setting to “reinforce what you learn and helping you to remember how to do it” and hence, maintain achieved benefits without *slip back into bad habits*:

The physio tells me that I'm walking too much on one side or the other side of my foot, and I do that, but I'm simply myself not aware of it, so if there was something that was just reminding me, that would be brilliant (FG2, F1)

Patients also discussed the advantages of having an ongoing data gathering of their movement function, especially when they would be away from their clinicians, and how this could prove beneficial in their subsequent visits in terms of management and planning ahead:

I mean it's like having a physiotherapist by your side so when you do go and see her, him or her, they've got all this, they've been there with you and so they can say, well, this is what you were doing, I was there. Not really, but sort of, because of the machine (FG3, F1)

In relation to how participants perceived the usefulness of objective data in the treatment decision-making process, it was felt that objective information would help them during the consultation by providing a clear explanation of their current status beyond their subjective description and perceptions:

So having something which can be more precise rather than you trying to explain is I think a very attractive step forward really because it gives proper data rather than your understanding of what it is you're doing (FG4, F3)

This would also provide clinicians with extra information to tailor treatment to each patient:

Help the physio to give you the best exercises which are geared just for your needs (FG1, F1)

On a more personal level, there were also thoughts that the use of the technology could help patients to make more effective use of their time during the rehabilitation process:

I think it could also save the need to attend a hospital, doctor or physio appointment, if the data could be transmitted using the internet, downloaded and transmitted that way, because I know they do it for, particularly in remote communities. They do ECGs and all that remotely. The data could be sent to your healthcare professional, and they could say, yeah, that's fine, we don't need to see you or I think perhaps we'd better have a, you'd better come and see us (FG2, F3)

Analogously, the accessibility of objective information on movement function could speed up the assessment process:

A quicker, less pain, hopefully, at the end you will have more information, your problem will be sorted out quicker, whereas if you're going the traditional route you're talking about months sometimes (FG4, F3)

Participants also highlighted how the use of technology could, in addition to saving time, reduce costs for themselves:

I'd say cost and/or time because, time is a personal cost and you can spend hours waiting for X-rays, waiting, going to see physios, waiting, going to see your GP who spends ages for his letter before it gets to the consultant who's away for three months who when you finally, all of that is time and it's tedious and it's phone calls and it's, so I think time and cost (FG4, F2)

It would reduce healthcare system costs as well:

If a patient, I don't know what it costs, is it £600 a visit or something? Well, that's a lot of money saved (by healthcare system) if you're just cutting out a few visits, money that could be used for everybody's benefit (FG2, F3)

The cost of the actual system, although with suggestions of being reasonably inexpensive, was not seen as a limitation to its adoption:

Well if it's going to help me I don't care what it costs, to be honest. It's to my benefit. What's my health worth to me? (FG3, M1)

## Discussion

This study investigated patients’ preferences in relation to wearable technology and how they envisaged its optimal design in relation to its use. Overall, patients showed a positive response towards the use of technology within a rehabilitation context and recognised the benefits that they could obtain from its use.

The main determinants for acceptance of a wearable system were identified in its appearance and the comfort in wearing it. Design requirements were discussed in detail. Among these, patients expressed the necessity for a wearable system to be small, stable, lightweight, and discrete to enable them to wear their usual outfit with no constraints and no identity intrusion. In this regard, integration into a pair of leggings for daily use was discouraged. As for *how long* to wear it, all patients agreed to not wearing the system over night and most advocated its use during exercising. However, a few patients recognised the benefits of wearing it while out and about. This is in line with clinicians’ and researchers’ beliefs of the importance of data in real-life scenarios of daily activities and most of all, the need of objective data over self-reported measures of movement function to tailor and optimise treatment provision.^1^ ^19^ *How* and *when* patients will wear the system will be the key in identifying the functional variables (eg, knee range of motion, distance, step numbers, exercise performance) that could be acquired over the defined period and the mode of feedback (eg, real-time, progress plot) that will be useful for clinical applications, encompassing patient and clinician use.

With regard to *how* to use the wearable technology, participants recognised the benefits of using the device as a system for supporting themselves over their rehabilitation course. In particular, they indicated the usefulness of the system in monitoring their movement function, encouraging and motivating them towards exercising, providing virtual supervision, correcting their movement and as a new means of communication with health professionals. The fact that participants perceived the use of technology as an incentive to adhere to rehabilitation regimes supports current trends of finding effective approaches to motivate patients into exercising and ensure continuity of rehabilitation therapy in the long term to maximise its success.^20–22^ The use of wearable technology could offer a novel way to deliver rehabilitation for patients with OA at home while ensuring virtual supervision via aerial data sharing with clinicians. This, however, is only possible if new developed systems align with users’ preferences. Patients could also envisage a more effective use of their time and money derived by the additional information available from the system. All participants agreed that the information collected would give them more control over their condition and permit their clinicians to be more informed about their problems, thus facilitating individualised treatment planning.

Our findings suggest the need for a sensor solely targeted towards exercise guidance and assessment, and a sensor that could also allow unobtrusive monitoring in everyday environments. This implies different design and technical specifications need to be taken into account in the developmental stage along with the choice of clinical outputs. Issues of data storage, transmission, visual feedback—either in real-time or in the form of periodical report, accuracy, and clinical utility of the outputs measured all need to be fully addressed for the technology to prove useful and meet users’ requirements. Moreover, some participants also suggested the use of haptic feedback to correct their movement; this will add another challenge to developers. Vibration motors used for gait retraining over a 6-week period have been shown to be effective in reducing knee adduction moment and pain in a small cohort of knee OA patients, with results retained at 1-month follow-up.^23^ Therefore, there is great potential to use technology to improve outcomes in patients with OA of the knee. Such potential will increase if the findings from our study are considered in finalising the wearable technology along with clinical trials to establish optimal OA treatment prescription, delivery and management in the long term. The use of technology has been speculated to lead to new evolving patient-driven healthcare models, where the information available will not only empower the patients in being more engaged on their conditions, as our participants also revealed, but will also influence how clinicians deliver treatment and use the information collected to make new recommendations.^24^

The design requirements identified agree with those outlined in the current literature but address the perceived need in the literature to explore patients’ preferences in relation to wearable technology.^14^ Previous studies reported on health-related information acquisition via portable devices or home sensors rather than functional movement monitoring,^14^ ^25–28^ considered a population other than patients with OA,^14^ ^26^ ^28–31^ identified design requirements without asking patients for their preference,^16^ or used a questionnaire approach rather than an open debate. It is known that focus groups permit a deeper investigation of participants’ views.^14^ ^15^ There have been brief reports on patients’ experience after using a particular device, but these focused more on technical aspects.^32^ ^33^ In contrast to previous studies, the cost of the system itself was not seen as a factor to limit adoption since it was perceived that the benefits outweighed the costs.^14^ ^34^ Our patients could foresee a long-term advantage in using the device as a way to employ National Health Service (NHS) resources more effectively for ‘*everybody's benefit*’. Moreover, beyond the design requirements of the device, we also asked questions that were different from previous studies, including how patients felt about the use of a wearable system for their condition and how they would fit it into their routine and use it for their benefit. This is where commercial wearable devices fall short: these are well-designed in terms of aesthetics and usability, but are mainly designed to track fitness, mostly in healthy people and sports-addicts; these lack the clinical interlink in tracking functional outcomes and lack the understanding of specific clinical population needs.^31^ Whereas these devices could be used as an incentive to keep people active, patients asked for direct measures of knee function and performance rather than general activity levels or calories burned, which are more commonly obtained from devices currently on the market. Moreover, when used in a randomised clinical trial, one of these market devices did not improve activity levels in a control group,^35^ highlighting once more the need for effective solutions to help patients adhere to rehabilitation regimes and address users’ needs in relation to technology. For the development of our technology, we adopted a user-centred and holistic approach, previously recommended,^36^ to enrich the technology value and meet user requirements, thus facilitating future uptake.

Although we focused on an OA population, our findings are transferable to other conditions where pain and joints movement are the focus of rehabilitation. Studies involving chronic pain have highlighted similar outcomes.^28^ ^31^ However, despite the main concepts being similar, the clinical outputs may change; therefore, each technology should be tailored to each clinical presentation, adhere to clinical guidelines in the assessment of patient performance, and consider emotional states associated with the condition when defining goals.^31^ For example, our device could permit monitoring of performance-based tests that are recognised by the clinical community as a measure to monitor the movement function of patients with OA.^37^ Similarly, these could be adjusted to other conditions.

There were no restrictions in participants’ recruitment with regards to their background, financial capacity and ethnicity so as to provide a general view of our population. Our sample represents a typical OA group, aged 45 years and over, with the majority being female.^38^ However, participants were only recruited within the London area and were unable to try out the devices, which are limitations of the study.

In conclusion, this paper presents a qualitative study aimed to investigate patients’ preferences of the design and usage of wearable technology. Outputs from this study should guide the design of wearable technology to maximise user acceptance. Participants were positive and supportive for the use of wearable technology as a rehabilitation aid. This should be taken into account in the clinical environment, and encourage developer and researcher to address patients requirements so as to accelerate clinical translation and hence, enhance patients’ benefits.
